# *In-silico* target prediction by ensemble chemogenomic model based on multi-scale information of chemical structures and protein sequences

**DOI:** 10.1186/s13321-023-00720-0

**Published:** 2023-04-23

**Authors:** Su-Qing Yang, Liu-Xia Zhang, You-Jin Ge, Jin-Wei Zhang, Jian-Xin Hu, Cheng-Ying Shen, Ai-Ping Lu, Ting-Jun Hou, Dong-Sheng Cao

**Affiliations:** 1 Xiangya School of Pharmaceutical Sciences, Central South University, Changsha, 410013 Hunan People’s Republic of China; 2 Department of Pharmacy, Jiangxi Provincial People’s Hospital, The First Affiliated Hospital of Nanchang Medical College, Nanchang, 330006 Jiangxi People’s Republic of China; 3 The First Hospital of Hunan University of Chinese Medicine, Changsha, 410007 Hunan People’s Republic of China; 4 Departments of Biomedical Engineering and Pathology, School of Basic Medical Science, Central South University, Changsha, 410013 Hunan People’s Republic of China; 5 Institute for Advancing Translational Medicine in Bone and Joint Diseases, School of Chinese Medicine, Hong Kong Baptist University, Hong Kong SAR, People’s Republic of China; 6 Innovation Institute for Artificial Intelligence in Medicine of Zhejiang University, College of Pharmaceutical Sciences, Zhejiang University, Hangzhou, 310058 Zhejiang People’s Republic of China

**Keywords:** Target prediction, Chemogenomic, XGBoost, Ensemble model

## Abstract

Identification and validation of bioactive small-molecule targets is a significant challenge in drug discovery. In recent years, various *in-silico* approaches have been proposed to expedite time- and resource-consuming experiments for target detection. Herein, we developed several chemogenomic models for target prediction based on multi-scale information of chemical structures and protein sequences. By combining the information of a compound with multiple protein targets together and putting these compound-target pairs into a well-established model, the scores to indicate whether there are interactions between compounds and targets can be derived, and thus a target prediction task can be completed by sorting the outputted scores. To improve the prediction performance, we constructed several chemogenomic models using multi-scale information of chemical structures and protein sequences, and the ensemble model with the best performance was used as our final model. The model was validated by various strategies and external datasets and the promising target prediction capability of the model, i.e., the fraction of known targets identified in the top-k (1 to 10) list of the potential target candidates suggested by the model, was confirmed. Compared with multiple state-of-art target prediction methods, our model showed equivalent or better predictive ability in terms of the top-k predictions. It is expected that our method can be utilized as a powerful computational tool to narrow down the potential targets for experimental testing.

**Graphical Abstract:**

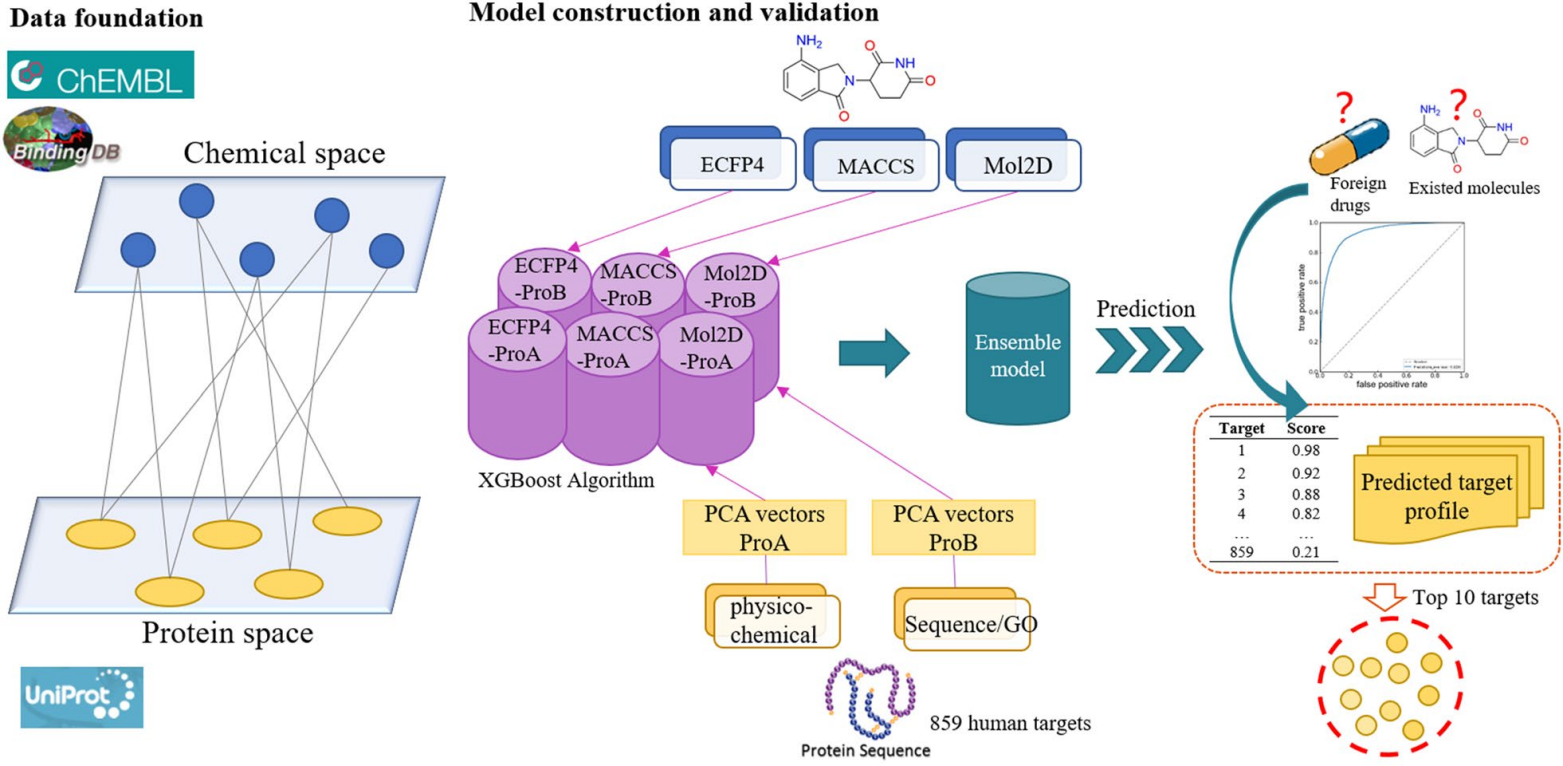

**Supplementary Information:**

The online version contains supplementary material available at 10.1186/s13321-023-00720-0.

## Introduction

It is estimated that 52% of clinical phase II failures are primarily due to insufficient efficacy, in which most are caused by poor targeting or unfavorable off-target effects [1, 2]. Apparently, identification of the potential targets for drug candidates in the early stages of drug discovery may reveal their adverse side effects, thereby reducing the attrition rate in clinical trials. Moreover, traditional drug discovery primarily followed the ‘one-compound, one-target, one-disease’ diagram, implying that a drug is designed to modulate a single target for a specific disease. But it is well known that most drugs bind to multiple targets, a general phenomenon known as polypharmacology [3]. The interactions between these secondary targets and drugs may be responsible for unexpected off-target effects, which usually induce unfavorable side effects but may also provide more opportunity for drug repositioning. For example, Sildenafil, the first selective type 5 phosphodiesterase (PDE_5_) inhibitor for the treatment of angina pectoris, has been repurposed for the treatment of penile erectile dysfunction (PED) and pulmonary hypertension [4]. Other notable drug repositioning examples include Memantine [5], Buprenorphine [6], Requip [7, 8], Colesevelam [9] and so on. Nowadays, due to the great difficulty and financial strain of drug discovery, identifying new indications for old drugs is informed the best way to bring a drug to market [10].

With the development of experimental techniques, protein targets can be identified by chemical proteomics methods such as affinity chromatography and activity-based protein profiling. In such experiments, modified or labelled compounds would specifically bind to proteins, and then the related protein targets can be precipitated or traced [11–18]. However, the modification and labelling of the key functional groups of the query compound may hamper compound-protein interactions. Moreover, these experimental approaches are labor-intensive, time-consuming and costly, and may suffer from high false-positive rate. Alternatively, driven by massive bioactivity data deposited in public chemogenomic databases such as ChEMBL [19], DrugBank [20], and TTD [21], *in-silico* target prediction has shown promise in recent years. By screening a compound against a database, it is possible to identify potential target candidates for subsequent experimental validation [22–25].

Generally, computational target prediction methods are classified into two categories: structure-based and ligand-based. The former methods detect the possible targets based on the three-dimensional (3D) crystal structure information of proteins, focusing on docking a query compound either to a set of targets or mapping to the pharmacophores inferred from ligand-target complexes [26–29]. However, the necessity of the 3D structures of proteins makes these methods applicable to a small range. Moreover, the uncertain relation between bioactivities and physicochemical properties served for scoring and the insufficient accuracy of scoring functions also show their weakness. Differently, the latter methods, mapping targets through the insight of the similarities between two compounds based on the hypothesis that similar compounds are likely to have similar target-binding profiles, are approved to achieve better predictive performance [30–34]. The most common implementation is machine learning (ML), which is accomplished by combining multiple independent binary classifiers. Each binary classifier trains on ligand information (i.e., descriptors) associated with a target and then learn knowledge that can correctly map descriptors to the target [35–38]. However, as ML methods do not take any protein information into account, the interactions between targets and compounds have not been fully explored. More importantly, if the number or structural diversity of the ligands for some targets is insufficient, the mapping functions cannot be guaranteed and well established.

Recently, the chemogenomic methods, by combining the protein sequence information with the compound-target interaction data to prediction models, making up for some key information of interactions and increasing the number of ligands for some targets by sharing ligands with targets having similar sequences, offset some weakness of ML methods discussed above [39–41]. These methods utilize both ligand and target spaces to extrapolate the bioactivities of compounds. Typically, a vector of descriptors representing each compound-target pair is taken as the input, and the output is whether there is an interaction between a compound and a target [42–44]. What inspires us is that, given the characteristic of this approach, a target prediction task can also be completed through combining a basket of protein targets for a compound and putting these compound-target pairs into a model to yield predictions. The score of each compound-target pair represents the probability of the association between the compound and protein, and finally the top-ranked targets are regarded as the potential targets. But as we already known, the prediction performance of the methods for target prediction has not been systematically evaluated.

In this study we constructed several chemogenomic models by integrating two types of protein descriptors and three types of molecular descriptors. These models are equipped with good binary classification that can accurately differentiate the compound-target interactions with strong binding affinity from those with weak binding affinity. Driven by the fact that the more descriptors from different insights included in the models, the more excellent prediction will be derived, different ensemble ML models were established and fully assessed, and the best one was selected as the final prediction model. The target prediction performance of the models (i.e., the fraction of known targets identified in the top-k (1 to 10) prediction list) was validated by various strategies and external datasets. Statistically, 26.78% and 57.96% of the known targets were enriched in top-1 and top-10 of the prediction list according to the stratified tenfold cross validation, respectively, suggesting approximately 230-fold and 50-fold enrichments. When validated by the external datasets including natural products, more than 45% targets were enriched in the top-10 of the prediction list. Compared with multiple state-of-art target prediction methods, our model yielded equivalent or better predictive ability on the top-k predictions.

## Materials and method

### Dataset collection

**Fig. 1:**
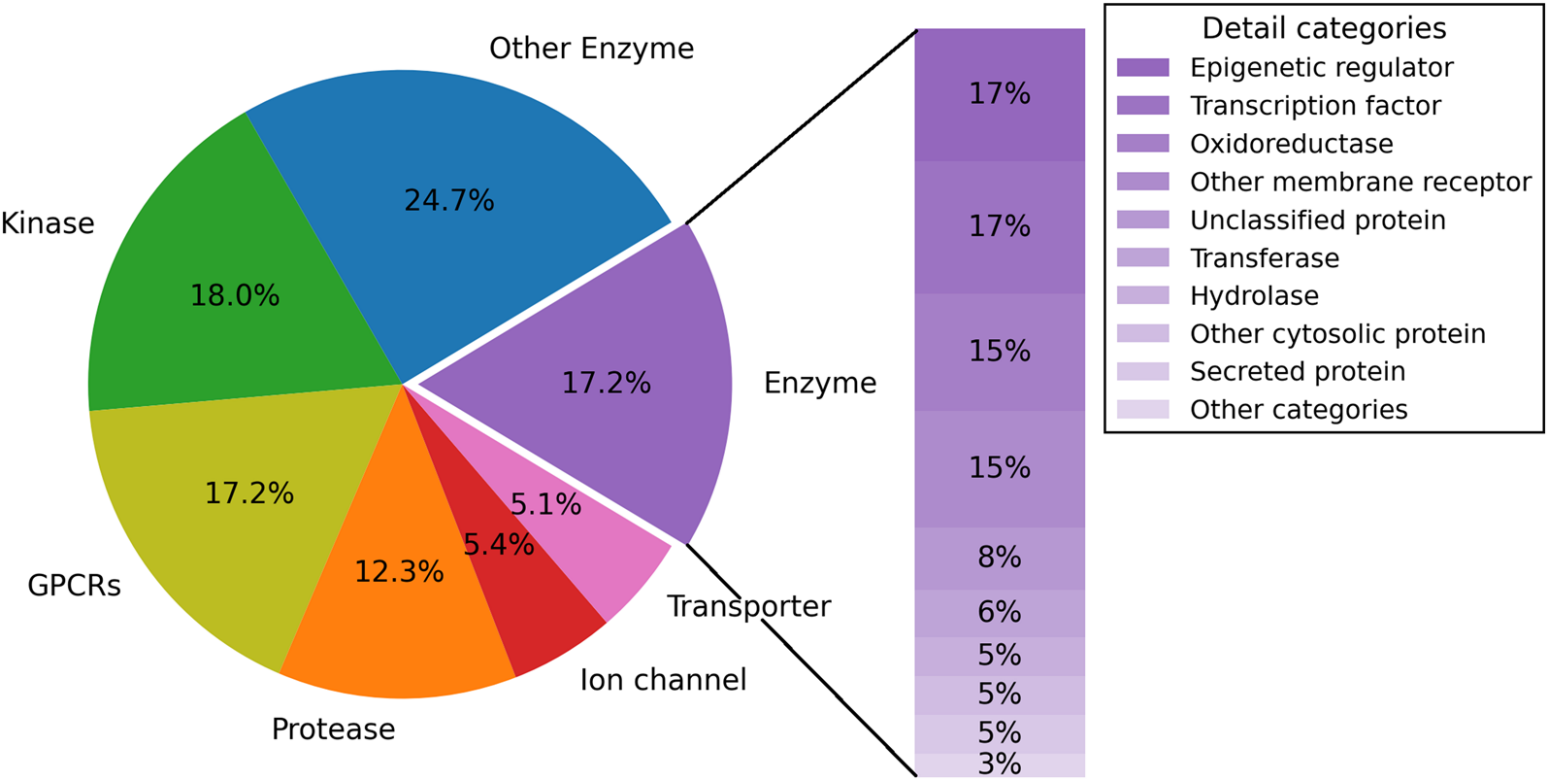
Category distribution of 859 targets for prediction

In this study, 859 human target proteins from the ChEMBL27 database [45] were collected for target prediction. Although some target proteins from other species also bind drug-like ligands, they were excluded because the prediction of targets against human species is our main focus. The collected targets mainly cover kinases, GPCRs, proteases, enzymes, and proteins from other detailed categories, among which 294 are FDA approved targets, 256 for clinical trial targets, 53 for patent targets, 236 for investigational targets and 20 targets documented in the literatures (Fig. 1). In addition, the target information including protein sequence and gene ontology (GO) terms with three subclass of biological process (BP), molecular function (MF) and cellular component (CC) was retrieved from the UniProt database [46] in order to facilitate the calculation of protein descriptors for constructing the prediction models. The detailed information of these 859 targets served for prediction can be seen in Additional file 1.

The entire dataset for modeling is composed of 153,281 compound-target interactions extracted from the Binding database v2020 [47] and the ChEMBL27 database [45], associated with 859 target proteins and 93,281 unique compounds. For each compound-target pair, its corresponding bioactivity data (*K*_i_) were extracted from these two databases. It is possible that multiple bioactivity data may be found for one compound-target pair due to the integration of different sources or literatures. A median of these bioactivity data was used for such pairs whose difference is below one magnitude, and those pairs whose bioactivity difference exceeds one magnitude were excluded. The detailed information of modeling data can be seen in Additional file 2.

**Fig. 2:**
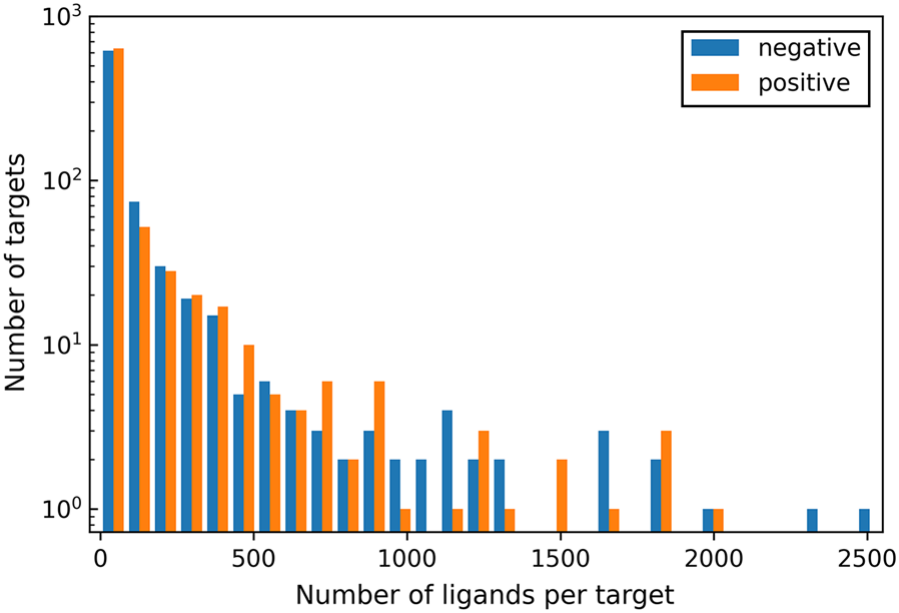
The distribution of the numbers of the positive samples and negative samples associated with each target

The *K*_i_ value of 100 nM was used as the threshold to tune the positive set (compound-target pairs with *K*_i_ ≤ 100 nM) and the negative set (compound-target pairs with *K*_i_ > 100 nM). Thus, the entire data set was firstly divided into 80,608 positive samples and 72,673 negative samples. Of these 859 targets, 549 had 10 or more known data points, 240 had more than 100 data points and 37 had more than 1000 data points. In more detail, 380 targets had 10 or more positive samples (namely active compound-target interactions), 145 targets had more than 100 positive samples, and 17 targets had more than 1000 positive samples (Fig. 2). On average, each target had 173.6 data points and 93.8 positive samples, the maximum numbers of the data points and positive samples of a target (carbonic anhydrase 2) were 3,351 and 2,013, respectively.

### Chemical structure and protein sequence representation

The descriptors used to represent and describe data decide the application range and success of a ML model. Structural descriptions under different levels sketch different compound/protein behaviors and provide diverse clues to inferring compound-target interactions. One-sided descriptors may not contain enough features to fully characterize the chemical and biological spaces of the data, provided the occurrence of “activity cliff” which presents pairs of compounds with high structural similarity but unexpectedly large activity (or property) difference. As a supplement, this gap may be captured by other types of descriptors. Therefore, to fully represent the comprehensive target-ligand interaction space, compounds were represented by three types of descriptors and proteins were described by three-level characterizations involving physicochemical properties, protein sequences and gene ontology (GO) terms.

For compound representation, three different molecular descriptors without the requirements of the three-dimensional conformations were used: (1) 188 Mol2D descriptors derived from the article proposed by Dong et al*.*, including 30 molecular constitutional descriptors, 25 topological descriptors, 44 molecular connectivity indices, 7 kappa shape descriptors, 21 Basak descriptors, 25 charge descriptors, and 60 MOE-type descriptors [48, 49]; (2) Extended Connectivity Fingerprint with a bond diameter of 4 (ECFP4), a class of 1024 bit circular fingerprints developed specifically for SAR modeling [50]; (3) MACCS fingerprints recording the occurrence of 166 predefined substructures reported to effectively encode molecular structure [51]. The above descriptors were calculated using MOE [52], ChemDes [53], ChemoPy [54], PyBioMed [48], and PyDPI [55].

The used protein descriptors were classified into two parts: protein type A (ProA) and protein type B (ProB). Protein sequences from the Uniprot database were used as the source for calculation. ProA was designed to execute the computation of seven physicochemical descriptor groups including amino acid composition descriptors, autocorrelation coefficient descriptors, CTD (composition, transition, and distribution) descriptors, conjoint triad descriptors, quasi-sequence-order descriptors, pseudo-amino acid composition descriptors, and proteochemometrics descriptors [48]. In order to reduce the model load, the multivariate descriptors (more than 50 dimensionality) from different sub-groups are projected to a lower-data space (50 dimensionality) from its most informative viewpoint by principal component analysis (PCA) [56]. Thus, each protein is described by 762 ProA descriptors. ProB is the descriptors derived from similarity matrix, which records the similarity between each pair of 859 protein pairs, including protein sequence similarity and the GO term similarity. Technically, the sequence similarities between each pair of proteins were calculated using the Resnik algorithm and GO semantic similarity measures including BP, MF and CC were obtained using the BLOSUM62 algorithm. In this manner, four similarity matrices of 859 × 859 were obtained, and each row of the matrix is the descriptors for each protein [57, 58]. Through PCA as before, ProB descriptors of each sample were reduced to 200 (4 × 50) dimensionality [56]. The detailed information of the descriptors and the percentage of explained variance (%VAR) of PCA were shown in Additional file 3. Here, the introduction of the matrix-derived descriptors limits the target prediction application of the models to these 859 targets [43, 59–61]

In this work, we employed the chemogenomic approach to encode the compound-target interactions by using both compounds and target proteins. An interaction can be efficiently represented by simultaneously considering the structural information from this compound and this protein. Through the combination of the structures from related compound and related protein (i.e., six combined descriptors of ECFP4-ProA, ECFP4-ProB, Mol2D-ProA, Mol2D-ProB, MACCS-ProA, and MACCS-ProB), each interaction sample (positive or negative) is finally characterized as a 1786, 1044, 950, 388, 928, 366 dimensional vectors, respectively.

### Machine learning methods

The extreme gradient boosting (XGBoost) algorithm was employed to construct the classification models [62]. XGBoost is an efficient and scalable implementation of the gradient boosting framework, and it provides insights on cache access patterns, data compression, and fragmentation. XGBoost develops the model in a sequential stage-wise fashion like other boosting methods do, and generalizes them by allowing optimization of an arbitrary differentiable loss function [63–65]. It has been regarded as a new generation of ensemble learning algorithms, which has become the winners for several ML competitions in recent years [66].

In the implementation, Konstanz Information Miner (KNIME) [67], a platform integrating data processing, data analysis, data exploration and Python package [68], was applied to construct models. The main hyperparameters, including learning rate (eta), the maximum depth of a tree (maximum depth), the minimum loss reduction required to make a further partition on a leaf node of the tree (gamma) and the number of models to train in the boosting ensemble (boosting rounds), were optimized by using the grid search method and the stratified tenfold cross-validation.

### Performance evaluation

The primary task of the model is to distinguish compound-target interactions with strong binding affinity from those with weak binding affinity, namely binary classification. Only when the model is equipped with satisfied binary classification performance, the target prediction performance can be guaranteed because it requires not only the binary classification capability of the classifier but also the ability to enrich the potential active targets of the compound at the top of the prediction list, namely early retrieval, typically the top 10 targets (top 0.1–1%) so that users are able to obtain a reasonable number of targets to be experimentally tested. Therefore, the binary classification performance of the model is firstly evaluated, and subsequently the target prediction ability is measured.

To ensure that the derived model has good generalization ability, the stratified tenfold cross-validation (CV) was used where the stratification process guaranteed that samples from each target were present in both the training and test dataset and samples from some targets which have a small quantity of ligands (≤ 5) were present only in the training datasets. By definition, the compounds or targets in the training set are called ‘known’, whereas those not existing in the training set are called ‘new’. Compared with the training set, there are two types of test set: (1) known compounds and known targets (intend to identify more possible targets for known active compounds); (2) new compounds and known targets (intend to identify targets for new compounds). Therefore, we conducted two levels of validation: pair-split validation and compound-split validation. As for the pair-split validation, the training and test sets were generated by randomly splitting the dataset according to the stratification. It measures the average performances of our models as the test datasets include both two types of pairs. As for the compound-split validation, it splits the compounds into 10 parts, and therefore the compound-target interactions associated with 1 out of these 10 parts were used as the test set and the interactions associated with the remaining 9 parts were kept in the training set. It assumes the situation where we want to detect the targets for external compounds. In order to evaluate the robustness of the model, the stratified tenfold CV was repeated 50 times and the obtained mean values and variance values were used to quantify the performance.

In the assessment, the binary classification performance of compound-target interactions was evaluated by several commonly used statistical parameters: true positives (TP), false negatives (FN), true negatives (TN), false positives (FP), the overall prediction accuracy (ACC = (TP + TN)/(TP + TN + FP + FN)), the prediction accuracy of the positive set (Sensitivity, SE = TP/(TP + FN)), and the prediction accuracy of the negative set (Specificity, SP = TN/(TN + FP)). Besides, the receiver operating characteristic (ROC) curve was plotted, and the area under the receiver operating characteristic curve (AUC) was used to assess each of the models.

The target prediction performance was verified by the recall rate, namely the fraction of known targets identified in the top k of the prediction list. For each compound to be predicted in the test set, the features from its compound descriptors combined with 859 protein targets, namely 859 compound-target integrated descriptors (i.e., six combined descriptors of ECFP4-ProA, ECFP4-ProB, Mol2D-ProA, Mol2D-ProB, MACCS-ProA, and MACCS-ProB), were inputted into the corresponding prediction model and then 859 compound-target interaction scores could be outputted. The targets ranked top-k of the prediction list are recognized as the potential targets, whereas the other targets are assumed inactive. An arbitrary cutoff of k (1–10) predictions was feasible number of protein targets that could be screened and differences in classifier performance after this cutoff will be missed. The recall rate is relatively harsh as it requires a classifier to have placed a correct target for a compound in the top 0.1%-1% of the lists but it gives an indication for the practicability of a model for target prediction.

## Results and discussion

### Compound-target interactions can be accurately predicted from integrated features

Our first concern in this study is to construct a predictive model that can accurately differentiate compound-target interactions with strong binding affinity from those with weak binding affinity. To represent compound-target interactions, we used a chemogenomic framework. In brief, an interaction is represented by simultaneously considering the structure content from this compound and this protein. Thus, each interaction sample (positive or negative) is finally characterized by a fixed dimensional vector by combining the structural content from the related compound and protein. Each of these factors can be considered as a separate coordinate spanning a multidimensional space, and in this sense a compound-target interaction is an event in this type of multidimensional space.

**Fig. 3:**
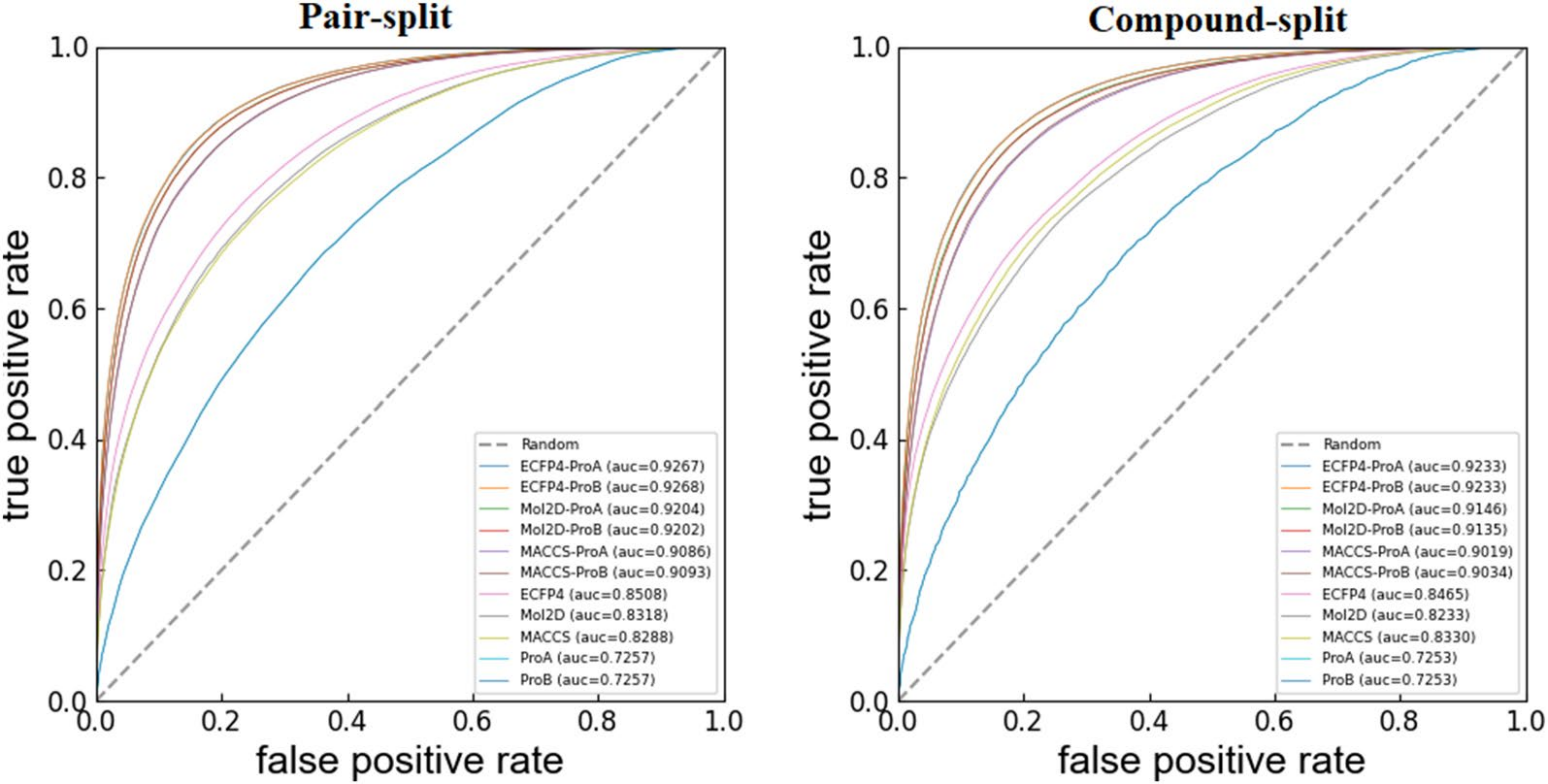
ROC curves of models derived from different descriptors (integrated or separated groups) on the stratified tenfold CV

Firstly, the classification performance of compound-target interactions was evaluated. The statistics of the predictions on the stratified tenfold CV are summarized in the “Integrated” rows of Table 1. The ROC curves are shown in Fig. 3.

**Table 1:** Statistical results of the models derived from different descriptors (integrated or separated groups) on the stratified tenfold CV

Descriptors		Pair-split				Compound-split			
		ACC	SE	SP	AUC	ACC	SE	SP	AUC
Integrated	ECFP4-ProA	83.96 ± 0.12	85.74 ± 0.11	82.00 ± 0.17	92.67	83.58 ± 0.06	85.35 ± 0.09	81.65 ± 0.09	92.33
	ECFP4-ProB	83.99 ± 0.16	85.87 ± 0.12	81.91 ± 0.22	92.68	83.55 ± 0.05	85.41 ± 0.08	81.51 ± 0.06	92.33
	Mol2D-ProA	82.11 ± 0.09	84.68 ± 0.10	79.29 ± 0.11	92.04	81.47 ± 0.05	83.95 ± 0.08	78.69 ± 0.03	91.46
	Mol2D-ProB	82.17 ± 0.13	84.85 ± 0.10	79.23 ± 0.18	92.02	81.59 ± 0.07	84.17 ± 0.07	78.75 ± 0.11	91.35
	MACCS-ProA	82.89 ± 0.25	85.00 ± 0.21	80.53 ± 0.34	90.86	82.24 ± 0.05	84.23 ± 0.08	80.04 ± 0.08	90.19
	MACCS-ProB	82.83 ± 0.27	85.02 ± 0.20	80.40 ± 0.33	90.93	82.09 ± 0.07	84.16 ± 0.11	79.81 ± 0.09	90.34
Separated	ECFP4	74.99 ± 0.03	76.21 ± 0.04	73.66 ± 0.09	85.08	75.28 ± 0.09	77.53 ± 0.12	72.76 ± 0.18	84.65
	Mol2D	74.09 ± 0.06	76.73 ± 0.07	71.17 ± 0.07	83.18	73.61 ± 0.07	76.99 ± 0.13	69.85 ± 0.16	82.33
	MACCS	72.83 ± 0.07	75.30 ± 0.09	70.12 ± 0.08	82.88	72.94 ± 0.08	76.45 ± 0.08	69.03 ± 0.18	83.30
	ProA	66.20 ± 0.01	72.51 ± 0.09	59.22 ± 0.13	72.57	66.21 ± 0.03	72.41 ± 0.20	59.34 ± 0.22	72.53
	ProB	66.21 ± 0.03	72.53 ± 0.08	59.20 ± 0.13	72.57	66.21 ± 0.03	72.44 ± 0.12	59.34 ± 0.15	72.53

From Table 1, both the pair-split and compound-split models performed well with an average ACC up to 0.81 and an average AUC up to 0.90, and the low standard deviations obtained from the 50 repetitions of the model shows the robust predictive performance of the models. These results above indicated that our models built with the six integrated descriptor groups and XGBoost algorithm could effectively distinguish the compound-target interactions with strong binding affinity from those with weak binding affinity. Unsurprisingly and reasonably, the performance of the compound-split validation is slightly worse than that of the pair-split validation (e.g., Mol2d-prob model ACC: 82.11 vs. 81.47) since these two strategies simulate different situations that actual predictions may encounter where the former means the prediction of brand-new “new” compounds while the latter additionally includes the prediction of “known” compounds whose associated compound-target interaction(s) in the training set may provide prediction clues. The statistical values of the models built on the individual descriptor group were as follows in a decreasing order: ECFP4-ProA > ECFP4-ProB > Mol2D-ProA > Mol2D-ProB > MACCS-ProB > MACCS-ProA. The model utilizing the ECFP4-ProA descriptors yielded the best performance, with ACC = 0.832 and AUC = 0.913.

The chemogenomic approach, aiming at integrating the chemical space with the genomics space, is demonstrated to be strikingly helpful for representing compound-target associations. A demonstrable feature of our approach is that the information from compounds and targets were integrated to represent compound-target associations. We assume that compound-target interactions can be determined by the structural features from compounds and targets, which comprise of a pharmacological space. To demonstrate the reliability of our assumption, we re-established our model using only the structural information from a single space (i.e., chemical space or genomics space), that is, models are constructed only using the compound features or protein features, respectively. The statistics of these models on the stratified tenfold CV were summarized in the “Separated” rows of Table 1. The ROC curves of the re-established models were shown in Fig. 3.

As can be seen from Table 1, the models with the compound features or protein features provided noticeably inferior predictions. The comparison between the models with the separated features and those with the integrated features sufficiently indicates that the structural information from compounds and targets contributes to the discrimination of compound-target associations cooperatively. Somewhat surprising, our comparison also illustrated that the features from compounds seem to be more predictive than those from target proteins.

### The ensemble model performs well than individual models

**Fig. 4:**
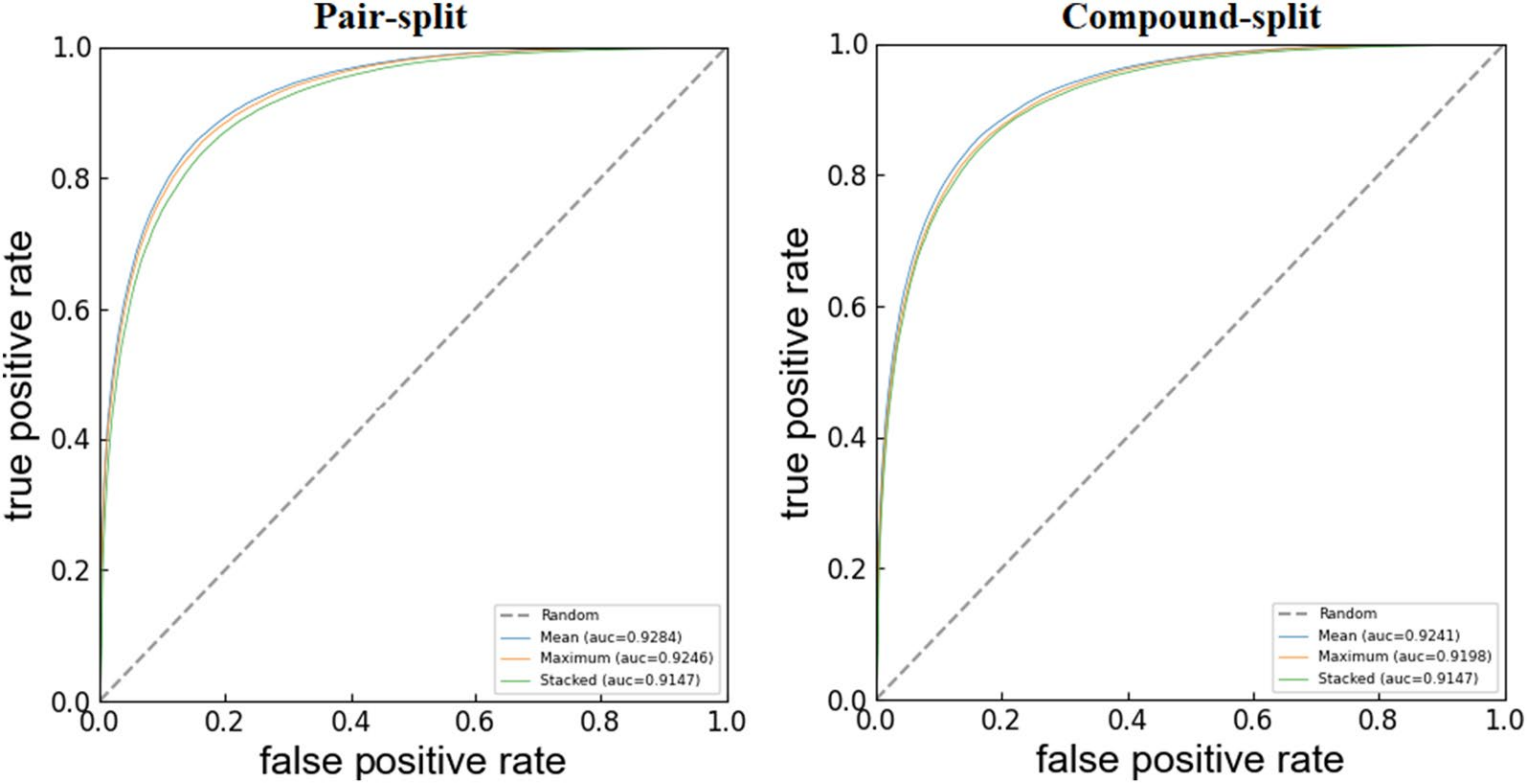
ROC curves of three ensemble models on the stratified tenfold CV

Due to the different strengths in compound-target interaction prediction caused by different descriptor groups, we attempt to improve the prediction performance through their combination. We built three types of ensemble models by averaging the predictions given by the six individual models (Mean) [69], taking the maximum value given by the six individual models (Maximum) and obtaining new scores using the stacked models reported by Nicholas (Stacked) [70]. The performance statistics are summarized in Table 2 and the ROC curves are shown in Fig. 4. The result shows that the ensemble model (Mean) yielded better predictive ability than any individual model, with the improved ACC of 0.01–0.1 and AUC of 0.01–0.1. It appeared that it could capture the relationship between compound-target interaction patterns and the interaction endpoint more efficiently than any individual model. Therefore, the ensemble model (Mean) was used as our final model and applied for the subsequent analysis.

**Table 2:** Prediction results of different ensemble models on the stratified tenfold CV

Methods	Pair-split				Compound-split			
	ACC	SE	SP	AUC	ACC	SE	SP	AUC
Mean	84.83 ± 0.16	86.96 ± 0.10	82.44 ± 0.24	92.84	84.41 ± 0.03	86.5 ± 0.04	82.13 ± 0.04	92.41
Maximum	80.73 ± 0.19	94.53 ± 0.08	65.39 ± 0.32	92.46	79.97 ± 0.05	94.63 ± 0.05	63.71 ± 0.08	91.98
Stacked	83.80 ± 0.23	85.07 ± 0.20	82.37 ± 0.30	91.47	82.93 ± 0.08	84.33 ± 0.08	81.40 ± 0.11	91.47

### Evaluation of the target prediction performance of our model

Under the premise of ensuring good classification performance of compound-target interactions, the target prediction performance of our model was then evaluated, which was the focus of our study that we attempted to verify whether our method could be expanded to the application of target prediction. For each compound to be predicted, a vector of 859 compound-target interaction scores could be outputted by our models and the targets with higher scores are considered as the target prediction result. Therefore, the target prediction performance was verified here using the recall rate, namely the fraction of the known targets identified in the top k of the prediction list. Undoubtedly, the performance improved with the increasing number of the picked targets. However, if the threshold of the number of selected targets is high, the number of the targets to be experimentally tested increases and thus the efficiency of the model application decreases. Inversely, if the threshold of the number of the selected targets is low, many targets recognized as inactive might be actually active. For practicality, approximately the top 1–10 targets out of the total 859 targets are proposed as the candidate targets.

The target prediction result on the stratified tenfold CV was showed in Table 3. The average recall rates of the top-1 and top-10 metrics for pair-split validation datasets were 28.54% and 59.50%, respectively, implying that there are 28.54% and 59.50% of known targets were enriched to the top-1 and top-10 of the ranked list by our model. Given that predictions were made among the 859 possible human targets, these recall rates of the top-1 and top-10 metrics correspond to approximately the 245-fold [28.54%/(1/859)] and 51-fold [59.50%/(10/859)] enrichment compared to random picking, respectively [30]. As for the compound-split validation datasets, the average recall rates of the top-1 and top-10 metric were 26.78% and 57.96%, respectively, which refer to approximately the 230-fold [26.78%/(1/859)] and 50-fold [57.96%/(10/859)] enrichments [30]. By the way, the targets to be correctly predicted evenly distributed across different target classes, which recognized the unbiased prediction performance for different target classes. This indicated that our model based on the ensemble chemogenomic approach could push true targets at the top of the ranking list and make some efforts to narrow down the potential targets to be tested.

**Table 3:** Recall rates of our model measured on the stratified tenfold CV datasets

	Top1	Top3	Top5	Top7	Top9	Top10
Pair-split	28.54 ± 1.22	43.92 ± 0.56	50.63 ± 0.46	55.00 ± 0.35	58.18 ± 0.28	59.50 ± 0.26
Compound-split	26.78 ± 0.12	42.80 ± 0.23	49.42 ± 0.22	53.59 ± 0.17	56.69 ± 0.17	57.96 ± 0.14

**Fig. 5:**
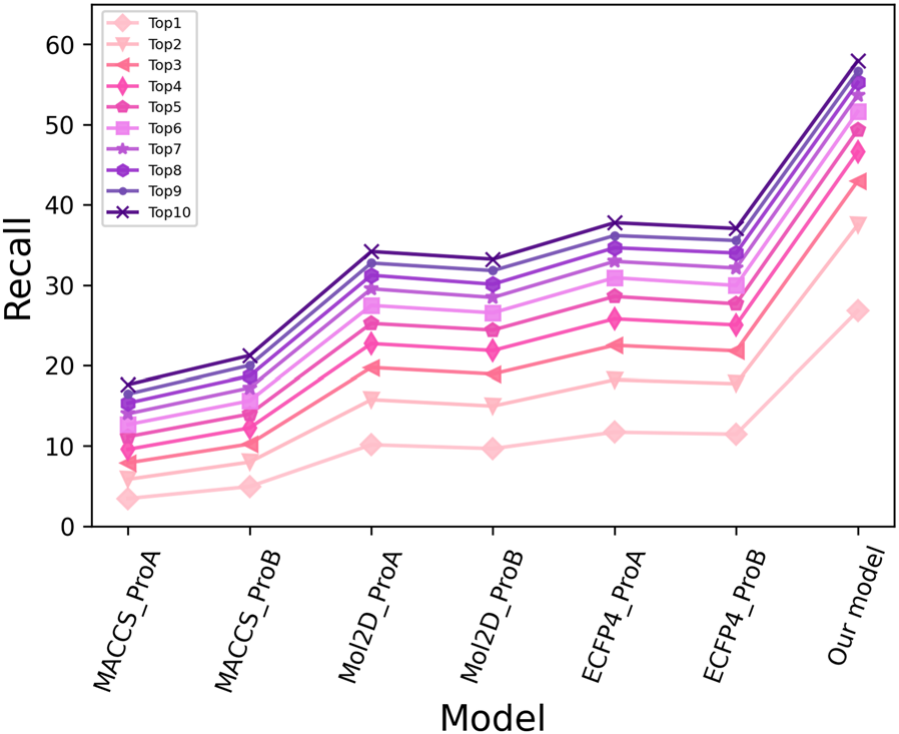
The recall rates of six individual models and our model within various top k values (k = 1–10) measured on the stratified 10-CV

To further verify whether our model had better target prediction performance than individual models based on various integrated descriptor groups, the prediction abilities of the individual models were prerecorded and compared with that of our model. As for the compound-split validation, the average recall rates of the top-k targets for diffident models on the stratified tenfold CV datasets, were plotted in Fig. 5. As shown in Fig. 5, the performance of the individual models was greatly inferior to that of our model. The recall value of each individual model for top 1 was lower than 20% even lower than 10%, while that of our model was 26.78%. The recall rate of top 10 for each individual model was lower than 40%, while that for our model was 57.96%. The recall values of the models in decreasing order were as follows: our model >  > ECFP4_Proa > ECFP4_Prob > Mol2d_Proa > Mol2d_Prob > MACCS_Prob > MACCS_Proa, which further illustrated the robustness and predictivity of our model based on the ensemble chemogenomic approach for target prediction.

### Target prediction performance for external test sets

To validate the generalization ability of our model on the external test dataset, we collected nonduplicated compound-target interactions with *K*_i_ less than 100 nM from the PDSP Ki (Psychoactive Drugs Screening Program Ki Database, https://pdsp.unc.edu/databases/kidb.php) and NPASS databases (Natural Product Activity & Species Source Database) [71] to evaluate the ability of the model. After compound filtering and preprocessing, we finally obtained 442 compounds with 778 compound-target interactions from the PDSP Ki database and 122 compounds with 181 compound-target interactions from the NPASS database. The two test datasets include 94 and 113 proteins, respectively. The detailed information of validation data can be seen in Additional file 4.

The target prediction results were shown in Table 4. For the compounds from the PDSP Ki database, 147 targets (out of 778) were ranked at the top-1 of the predicted target list, with a recall rate of 18.89%. The NPASS dataset obtained a recall rate of 8.84%, indicating that 16 targets (out of 181) were successfully predicted in the top-1 list. The performance gap between these two datasets might be explained by the fact that the enough knowledge about natural products didn’t be well learned by the model constructed by datasets mostly composed of synthetic compounds. However, whether for the PDSP Ki dataset or NPASS dataset, more than 45% targets were enriched in the top-10 of the predicted ranking list (a recall rates of 53.34% and 45.30% for PDSP Ki and NPASS for the top-10 prediction, respectively). Although the performance of these external datasets was fractionally inferior to that of the stratified tenfold CV, it highlighted the capability of our model to enrich active targets for different sets of compounds, even for natural products.

**Table 4:** The target prediction results of the external test sets

Top k threshold	PDSP Ki (778)		NPASS (181)	
	Count	Recall (%)	Count	Recall (%)
Top1	147	18.89	16	8.84
Top3	257	33.03	49	27.07
Top5	322	41.39	68	37.57
Top7	363	46.66	72	39.78
Top10	415	53.34	82	45.30

### Comparison with alternative approaches

Our model was compared with some state-of-the-art target prediction tools including SwissTargetPrediction (the updated 2019 version) [30], HitPickV2 [72], PPB2 [73], PPB [74] and TargetNet [36]. The comparison dataset was from the validation data from SwissTargetPrediction (from ChEMBL24) annotated as direct binders with the high activity (*K*_i_, *K*_D_, IC_50_ or EC_50_) < 1 nM, associated with 1,061 ligand-target interactions. The ligands present in our model were firstly removed from the model to rebuild a new one in order to avoid potential bias. The recall rate defined in this study was used in the comparison between our ensemble model and four web tools. As the performance of SwissTargetPrediction on this dataset is public and its statistics metric is different from that of our research, our model was re-measured by the same statistics metric to ensure fair comparison, that is, the fraction of compounds for which at least one known target was identified in the top-1 or top-15 of the prediction lists. The comparison results are listed in Table 5, and related detailed performance of individual methods can be seen in Additional file 5.

**Table 5:** Comparison results with alternative state-of-the-art prediction methods

TopK	^a^HitPickV2	^a^PPB2							^a^PPB	^a^TargetNet	^a^Our model	^b^Our model	^b^Swisstarget prediction
		NB(ECfp4)	NN(ECfp4) + NB(ECfp4)	NN(ECfp4)	NN(MQN) + NB(ECfp4)	NN(MQN)	NN(Xfp) + NB(ECfp4)	NN(Xfp)					
1	24.69	16.49	14.89	16.59	21.87	10.65	21.49	16.49	5.18	23.20	26.96	57.00	28.00
3	56.74	35.06	52.31	52.88	52.40	22.43	52.40	30.91	18.85	41.85	56.36	–	–
5	58.43	47.03	60.92	57.96	57.21	26.96	60.30	35.34	25.82	46.37	59.33	–	–
7	60.82	53.35	62.76	61.29	60.04	30.16	61.30	39.21	29.78	48.91	60.89	–	–
10	62.20	60.98	64.75	63.62	63.05	34.68	62.58	45.62	34.40	50.99	63.99	–	–
15	–	–	–	–	–	–	–	–	–	–	–	76.00	72.00

^a^Recall rate defined in our article (%); ^b^ the fraction of compounds for which at least one known target was identified in the top-1 or top-15 of the prediction lists (%)

The comparison results with HitPickV2, PPB2, PPB and TargetNet showed that our model performed better than any other method for the recall rate on top-1 predictions, including the popular HitPickV2 (Recall: 26.96% vs. 24.69%) and PPB2 method NN(MQN) + NB(ECfp4) (Recall: 26.96% vs. 14.89%). For the top-10 predictions, the performance of our model was better than those of all other models except PPB2 method NN(ECfp4) + NB(ECfp4) (Recall: 63.99% vs. 64.75%). The above results are very encouraging, especially since it is not clear whether the tested interaction pairs have been used in the construction of other models.

Comparison results with SwissTargetPrediction showed that for 360 molecules (72%), at least one of the experimentally known targets can be found among the predicted top-15 of SwissTargetPrediction, while for 379 molecules (76%), at least one of the experimentally known targets can be found among the predicted top-15 of our method. More importantly, our model detected at least one known target at top-1 prediction for 57.0% of ligands, with 28% for SwissTargetPrediction. These excellent results supported that our model is a strongly powerful target prediction engine to enrich active targets which may strongly bind/associate to compounds. It is expected to make some efforts for narrowing down the set of potential targets to be experimentally tested and to be of interest to the audiences for wider scientific community.

## Conclusion

Predicting targets of active compounds can augment modern drug discovery efforts in a range of applications, from the elaboration of molecular mechanisms and side-effect to the repurposing of existing drugs, and to designing novel drugs with lower toxicity and higher efficacy. However, identifying a direct target for active compounds remains a challenging task as a significant investment of time and resources is required for the experiments. Here, the chemogenomic modeling using the integrated features of compounds and proteins can be considered as a promising method for target identification.

We developed a model with the multi-scale information of chemical structures and protein sequences through the chemogenomic framework and the ensemble method to predict targets. It shows excellent target prediction statistics, which means to approximately 230-fold and 50-fold enrichment. The performance of our model was greatly superior to the individual models. When the model was validated by external datasets including natural products, more than 45% targets were enriched in the top-10 of the prediction list. Moreover, compared with multiple state-of-art target prediction methods, our model yielded equivalent or better predictive ability on the top-k predictions. In summary, the ensemble chemogenomic model constructed by us is expected to make some efforts for narrowing down the set of potential targets to be tested and speed up the process of the target identification.

For practicability, our model was public to facilitate predicting targets of interested compounds: https://github.com/SorchaYang00/Chemogenomic-Model. By inputting a compound, 859 targets can be hit and assigned by corresponding probabilities. Given the complexity of the interaction pattern between compounds and targets and derived numerous features/variables, neural network may provide progressive progress of the chemogenomic method for target prediction.

## Supplementary Information

**Additional file 1.** Targets that can be predicted by our model.

**Additional file 2.** The datasets supplied for model construction.

**Additional file 3.** The detailed information of the descriptors and the percentage of explained varianceof PCA.

**Additional file 4.** External test sets and corresponding validation results.

**Additional file 5.** Results and performance of our model and alternative state-of-the-art target prediction tools on the validation data from SwissTargetPrediction.

## Data Availability

All the datasets supporting the conclusion of this article are available in Additional files.
