# Supplementary material for: In-silico target prediction by ensemble chemogenomic model based on multi-scale information of chemical structures and protein sequences
Source: J Cheminform. 2023 Apr 23;15:48. doi: 10.1186/s13321-023-00720-0 (PMC10123967; doi:10.1186/s13321-023-00720-0)
Supplement: Supplementary file 3 — Additional file 3. The detailed information of the descriptors and the percentage of explained varianceof PCA. [file 13321_2023_720_MOESM3_ESM.pdf]

Protein Descriptors and Corresponding Principal Components

Class	Descriptor Group	Descriptor	Num.	PC Num.	%VAR
ProA	Amino Acid Composition	Amino acid composition	20	N/A	N/A
		Dipeptide composition	400	50	41.41
	Autocorrelation	Normalized Moreau-Broto	240	50	73.72
		Moran	240	50	66.20
		Geary	240	50	66.86
	CTD	Composition	21	N/A	N/A
		Transition	21	N/A	N/A
		Distribution	105	50	96.42
	Conjoint triad	Conjoint Triad	343	50	53.74
	Quasi-Sequence-Order	Sequence-order-coupling number	60	50	99.99
		Quasi-sequence-order descriptors	100	50	91.88
	Pseudo-Amino Acid Composition	Type I	50	N/A	N/A
		Type II	80	50	95.98
	Proteochemometric Descriptors	Principal components analysis (amino acid properties based)	175	50	66.02
		Factor analysis (amino acid properties based)	175	50	66.95
		Multidimensional scaling (amino acid properties based)	175	50	63.33
		BLOSUM matrix-derived descriptors	175	50	61.99
ProB	Matric-derived Similarity	Sequence similarity	859	50	37.52
		BP semantic similarity	859	50	97.31
		MF semantic similarity	859	50	98.38
		CC semantic similarity	859	50	99.37
